# Application analysis of omental flap isolation and modified pancreaticojejunostomy in pancreaticoduodenectomy (175 cases)

**DOI:** 10.1186/s12893-022-01552-9

**Published:** 2022-04-02

**Authors:** Shun Deng, Jianhong Luo, Yongzhong Ouyang, Jiangbo Xie, Zhuo He, Bo Huang, Fei Bai, Ke Xiao, Bin Yin, Jinfeng Wang, Biaoming Xu, Chaohui Zuo

**Affiliations:** Department of Gastroduodenal and Pancreatic Surgery, Translational Medicine Research Center of Liver Cancer, Laboratory of Digestive Oncology, The Affiliated Cancer Hospital of Xiangya School of Medicine, Central South University/ Hunan Cancer Hospital, Yuelu District, No. 283 Tongzipo Road, Changsha, 410013 Hunan China

**Keywords:** Modified pancreaticojejunostomy, Omental flap isolation, Pancreatic fistula, Postoperative complications, Radical pancreatoduodenectomy

## Abstract

**Background:**

To explore the application value of free omental wrapping and modified pancreaticojejunostomy in pancreaticoduodenectomy (PD).

**Methods:**

The clinical data of 175 patients who underwent pancreaticoduodenectomy from January 2015 to December 2020 were retrospectively analysed. In total, 86 cases were divided into Group A (omental wrapping and modified pancreaticojejunostomy) and 89 cases were divided into Group B (control group). The incidences of postoperative pancreatic fistula and other complications were compared between the two groups, and univariate and multivariate logistic regression analyses were used to determine the potential risk factors for postoperative pancreatic fistula. Risk factors associated with postoperative overall survival were identified using Cox regression.

**Results:**

The incidences of grade B/C pancreatic fistula, bile leakage, delayed bleeding, and reoperation in Group A were lower than those in Group B, and the differences were statistically significant (P < 0.05). Group A had an earlier drainage tube extubation time, earlier return to normal diet time and shorter postoperative hospital stay than the control group (P < 0.05). The levels of C-reactive protein (CRP), interleukin-6 (IL-6), and procalcitonin (PCT) inflammatory factors 1, 3 and 7 days after surgery also showed significant. Univariate and multivariate logistic regression analyses showed that a body mass index (BMI) ≥ 24, pancreatic duct diameter less than 3 mm, no isolation of the greater omental flap and modified pancreaticojejunostomy were independent risk factors for pancreatic fistula (P < 0.05). Cox regression analysis showed that age ≥ 65 years old, body mass index ≥ 24, pancreatic duct diameter less than 3 mm, no isolation of the greater omental flap isolation and modified pancreaticojejunostomy, and malignant postoperative pathology were independent risk factors associated with postoperative overall survival (P < 0.05).

**Conclusions:**

Wrapping and isolating the modified pancreaticojejunostomy with free greater omentum can significantly reduce the incidence of postoperative pancreatic fistula and related complications, inhibit the development of inflammation, and favourably affect prognosis.

## Introduction

Pancreaticoduodenectomy (PD) is a standard treatment approach for patients with malignant or benign diseases of the pancreatic head or the periampullary region [1]. Reconstruction of the digestive tract by PD is complicated and time- consuming, and has remained one of the most complicated and risky operations in abdominal surgery. Despite the continuous development and improvement of surgical techniques, complications cannot be completely avoided after PD. Postoperative pancreatic fistula (POPF) is the "Achilles' heel" of PD and a major driver of morbidity and mortality [2, 3]. Pancreatic fistula is an important complication that can occur after all types of pancreatectomy [4]. Postoperative pancreatic fistula, abdominal infection and delayed haemorrhage can affect each other [5].When pancreatic fistula occurs to a certain extent, it will lead to abdominal infection, which will further erode the gastroduodenal artery(GDA) and its surrounding vessels, leading to delayed haemorrhage and even death [6, 7]. The mortality rate of POPF can reach 20 to 50% [8]. How to effectively prevent POPF is a focus when implementing PD [9]. The occurrence of pancreatic fistula is affected by many factors, such as pancreatic texture, pancreatic duct diameter, intraoperative blood loss, postoperative pathological type, and pancreaticojejunostomy method [10, 11]. Among the above factors, only pancreaticojejunostomy can be controlled during the operation.

Pancreaticojejunostomy is an important part of pancreaticoduodenectomy. High-quality pancreaticojejunostomy can reduce the occurrence of anastomotic leakage to a great extent and accelerate patient recovery after surgery. The binding technique, tube-mucosa anastomosis and Blumgart technique are commonly used pancreaticojejunostomy methods in PD [12, 13]. In recent years, a variety of studies have focused on the relationship between the improvement of pancreaticojejunostomy methods and the incidence of pancreatic fistula, but there is currently no consensus. Studies have shown that the greater omentum has manyroles, such as promoting anticorrosion, and anti-infection effects, immune responses, secretion, and absorption of ascites [14–16]. The greater omentum can also regulate blood circulation in the gastrointestinal tract and transmit vascular endothelial growth factors, thereby accelerating the formation of new vessels at the anastomosis[17]. On this basis, our technical team removed the pediculated omental tissue and wrapped it around the pancreaticojejunostomy after modified end-to-side pancreaticojejunostomy, so that the serosal surface of the jejunum and the broken end of the pancreas could be covered more tightly and effectively, further avoiding the formation of dead space. Adhesion between omentum and anastomosis can improve the blood supply to the anastomosis. The purpose of this study was to analyse the changes in postoperative complications such as pancreatic fistula and biliary fistula after combining omental wrapping with modified pancreaticojejunostomy, to determine the significance of this technique in PD surgery. Specific reports are provided below.

## Materials and methods

### Preoperative data

This study was approved by the Ethics Committee of The Affiliated Cancer Hospital of Xiangya School of Medicine, Central South University/ Hunan Cancer Hospital as SBQLL-2021-051. There was no professional or financial connection between the study authors and any business entity.

The clinical data of 175 patients who underwent pancreaticoduodenectomy in the Affiliated Cancer Hospital of Xiangya School of Medicine, Central South University/Hunan Cancer Hospital from January 2015 to December 2020 were retrospectively analysed. The patients were divided into an omental wrapping and modified pancreaticojejunostomy group (GroupA, n = 86) and control group (GroupB, n = 89), according to whether omental wrapping and modified pancreaticojejunostomy were used. The inclusion criteria were as follows: 1. no cardiorespiratory insufficiency; 2. no recent inflammatory infection; and 3. no sign of distant metastasis on imaging, with surgical indications. Patients with locally advanced pancreatic cancer and pancreatic cancer with distant metastasis were excluded. There was no significant difference in sex, age, body mass index (BMI), preoperative liver function, preoperative albumin, preoperative bilirubin, pancreatic duct diameter, and postoperative pathology between the two groups. Preoperative C-reactive protein (CRP), interleukin-6 (IL-6), procalcitonin (PCT) and other inflammatory indicators were in the normal range and there were no significant differences between the two groups of patients. Before surgery, 16 patients (Group A) and 14 patients (Group B) with borderline resectable pancreatic cancer received 2 to 4 cycles of neoadjuvant chemotherapy with the FOLFIRINOX regimen through multidisciplinary therapy (MDT).

### Surgical operation and key points

**Fig. 1:**
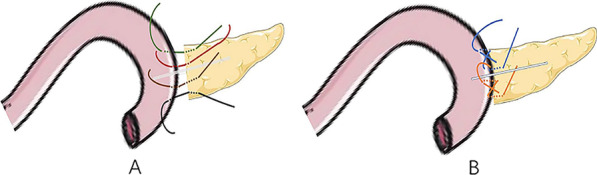
Modified pancreaticojejunostomy model diagram: **A** u-suture through the pancreas. **B** Pancreas and jejunum seromuscular layer "8" suture

Standard PD was performed in 175 cases, and regional lymph node dissection was performed routinely during the operation. During the operation, the hepatoduodenal ligament, celiac trunk, portal vein, common hepatic artery, proper hepatic artery, and superior mesenteric vein were skeletonized, and Child’s anastomosis was used to reconstruct the digestive tract (pancreaticojejunostomy, biliary intestine, gastrointestinal anastomosis). (1) Omentum wrapping and modified pancreaticojejunostomy group: After a Kocher incision was used to fully dissociate remove the lymph nodes, the mass was completely removed, and the digestive tract was reconstructed by Child's method. During pancreaticojejunostomy, the pancreatic stump was lifted first, and the back of the pancreas was separated. The small branches of the pancreatic arteries and veins were freed, from the pancreas by about 2 cm, the pancreatic duct, was located, a pancreatic duct drainage tube with 2–3 small holes on the bevel that matched the diameter of the pancreatic duct was inserted, and the distal jejunum was passed through the transverse colon system The hole in the avascular part of the membrane was pulled to the pancreas to perform end-to-side pancreatic jejunum anastomosis. A 4–0 absorbable thread was applied to fix the drainage tube. Approximately 2 cm from the upper and lower edges of the pancreas, 3–0 absorbable thread was applied to penetrate the pancreas from the ventral side to the dorsal side. A needle was used to place sutures in a "U" shape, and the vascular clamp, was removed so that the suture would not be knotted temporarily. If haemostasis of the pancreas stump was not satisfactory, a nonabsorbable mattress suture or 3–5 intermittent sutures of the pancreas stump were used. Approximately 1 cm from the severed end of the pancreas, a large needle with 3–0 Prolene was used to on the upper edge of the pancreas to suture the seromuscular layer of the pancreaticojejunostomy in an "8"pattern. A small hole in the jejunum corresponding to the pancreatic duct was cut to remove the pancreatic duct drainage tube. The drainage tube point into the jejunum and, was fixed with a full-layer purse-string suture with 4–0 absorbable thread; then,the pancreatic juice drainage tube was placed into the distal end of the jejunum loop. Finally, at the lower edge of the pancreas, a large needle with 3–0 Prolene was used to pierce the pancreas and jejunum serosa muscle layer with two stitches in an "8" pattern to tie the knot; that is, a modified pancreaticojejunostomy was used to complete the pancreaticojejunostomy. After completing reconstruction of the digestive tract, a section of pedicled omentum was selected, placed behind the pancreaticojejunostomy, and filled the area between the pancreatic stump and the jejunum. The upper boundary covered the horizontal line of the hepatogastric ligament, and the left boundary was the abdominal trunk arteries and omental sac. The right boundary was the right edge of the inferior vena cava, so that the flap wrapped, covered and protected the portal vein, common hepatic artery, GDA stump, superior mesenteric vein and superior mesenteric artery. The omentum of the pad was fixed to the hilar region of the liver and the hepatogastric ligament with 3–0 absorbable thread. (2) Control group: Child’s method was also used to reconstruct the digestive tract. After the pancreatic duct was located and the pancreatic duct drainage tube was placed, the jejunum after the ligament of Treitz was lifted, and the pancreas was severed to perform end-to-side pancreatojejunostomy. Next, 3–0 Prolene was used to suture the seromuscular layer of the jejunum to the back of the pancreas, a small hole was created in the jejunum, the pancreatic duct was inserted into the loop of the jejunum, and the pancreatic jejunum mucosa was sutured with 4–0 absorbable sutures. Using 3–0 Prolene sutures again, the seromuscular layer of the pancreas and jejunum were sutured continuously before the pancreaticojejunostomy was completed, and omental wrapping was not performed after pancreaticojejunostomy. The details are presented in Fig. 1. To decision to perform adjuvant treatment was based on the postoperative pathology report. Postoperatively, the R0 resection rates were 90.7% (Group A) and 86.6% (Group B). In Group A, 61 patients (70.9%) received adjuvant chemotherapy postoperatively. Fifty-eight patients (65.2%) in postoperative Group B were treated with adjuvant chemotherapy.

### Detection of inflammation specific protein marker

The CRP, IL-6, and PCT levels were monitored preoperatively and on the first, third, and seventh days postoperatively. Detection method: Five millilitres of fasting venous blood was drawn from patients in the morning and centrifuged at 3500 r/min for 15 min after standing, and the supernatant was retained. Serum CRP was measured by ELISA, and a CRP kit was purchased from Abcam Ltd. Serum IL⁃6 and PCT were detected by an electrochemical luminescence method. The kit was purchased from Roche Diagnostic Reagent (Roche Automatic Biochemical Analyzer).

### Diagnostic criteria for postoperative complications

Complications after PD mainly include pancreatic fistula, biliary fistula, intestinal fistula, intra-abdominal haemorrhage, anastomotic bleeding, delayed gastric emptying, and intra-abdominal infection. (1) Pancreatic fistula is the most serious complication with the highest mortality postoperatively. The currently recognized diagnostic criteria for pancreatic fistula issued by the International Research Group on Pancreas Surgery (ISGPS) in 2016 were adopted to identify pancreatic fistula [18]. The diagnostic criteria define pancreatic fistula as follows: amylase content of drainage fluid > 3 times the upper limit of normal more than 3 days after the operation. In these criteria, grade A pancreatic fistula was reclassified to biochemical fistula. (2) Biliary fistula (BF) occurs in 3 to 8% of patients [19, 20], and Stefano et al. [21] believe that BF is rare but can be life-threatening if it co-occurs with POPF. Biliary fistula is defined as abdominal biliary drainage with a bile component and a total bilirubin level above normal within 3 days after surgery [22, 23]. (3) Delayed intra-abdominal bleeding (PPH) was identified according to the 2007 ISGPS diagnostic criteria [24]: bleeding within 24 h after surgery was defined as early postoperative bleeding, and bleeding after 24 h after surgery was defined as delayed bleeding. (4)Delayed gastric emptying (DGE) was defined by the International Research Group on Pancreatic Surgery[25, 26]: patients who failed to return to a normal diet before the end of the first postoperative week were divided into three different grades (A, B, and C) according to the impact on the clinical course and postoperative management. (5)Surgical site infection (SSI) was defined according to the Centers for Disease Control (CDC) guidelines for the prevention of SSI [27]. (6)The criteria for removal of the abdominal drainage tube were as follows: amylase in drainage fluid < 1000 U/L, and drainage volume < 50 mL/d.

### Statistical analysis

Statistical software SPSS 26.0 was used for data analysis. Normality tests were performed for quantitative data, and the t test was used to compare data that followed a normal distribution; there data are expressed as x̄ ± s. Data following a non-normal distribution are expressed as the median (interquartile) and compared with non-parametric tests (Mann Whitney). Qualitative data are expressed as frequencies and were compared with the χ^2^-test. A logistic regression model and Cox proportional risk regression model were used for univariate and multivariate analyses. A two-tailed P < 0.05 was defined as statistically significant.

## Results

### Preoperative general parameters

There were no significant differences in the general parameters between Groups A and B (P > 0.05, Tables 1, 2). The pathological types in the two groups were as follows: Group A: 36 cases of duodenal papilla carcinoma, 7 cases of ampullary carcinoma, 22 cases of pancreatic head carcinoma, 7 cases of carcinoma in the lower segment of common bile duct, 6 cases of duodenal stromal tumours, 2 cases of pancreatic neuroendocrine carcinoma, 1 case of hilar cholangiocarcinoma and 5 cases of benign tumours; and Group B: 41 cases of duodenal papilla carcinoma, 3 cases of ampullary carcinoma, 32 cases of pancreatic head carcinoma, 4 cases of carcinoma in the lower segment of the common bile duct, 4 cases of duodenal stromal tumours, 2 cases of pancreatic neuroendocrine carcinoma and 3 cases of benign tumours. There were no significant differences in pathological type between Group A and Group B (P > 0.05, Table 3).

**Table 1:** Comparison of basic data between omentum liner and modified pancreaticojejunostomy group and control group

Factor		Group A (86 cases)	Group B (89 cases)	Statistical	P value
Gender	Male	49	60	2.029	0.1544
	Female	37	29		
Age		60.6 ± 9.6	57.6 ± 11.2	1.86	0.065
BMI		23.0 ± 1.9	23.1 ± 1.9	− 0.18	0.856
Albumin (g/l)	< 35	25	21	0.68	0.411
	≥ 35	61	68		
ALT (u/l)		18.33 (2.95 ~ 321.05)	19.41 (4.17 ~ 347.00)	− 0.52	0.958
Pancreatic duct diameter (mm)	≥ 3	42	52	1.618	0.203
	< 3	44	37		
Preoperative bilirubin level		44.135 (8.92 ~ 341.1)	30.175 (6.93 ~ 307.69)	− 0.807	0.419

**Table 2:** Comparison of preoperative inflammation index between two groups

Group	WBC (*10^9^/L)	CRP (mg/L)	IL-6 (pg/ml)	PCT (ng/ml)
Group A (n = 86)	7.23 ± 2.58	8.16 ± 3.52	6.27 ± 2.21	0.031 ± 0.012
Group B (n = 89)	7.44 ± 2.25	8.67 ± 2.78	6.33 ± 1.96	0.042 ± 0.007
Statistical	5.653	8.472	5.274	6.415
P value	0.581	1.255	2.327	0.227

*BMI* body mass index, *ALT* alanine aminotransferase, *WBC* white blood cell, *CRP* C-reactive protein, *IL-6* interleukin-6, *PCT* procalcitonin

**Table 3:** Comparison of postoperative pathology between omentum liner and modified pancreaticojejunostomy group and control group

Group	Pathological types						
	Duodenal tumor	Carcinoma of head of pancreas	Carcinoma of ampulla	Cancer of the lower segment of common bile duct	Mesenchymoma	Innocent tumour	Other tumors
Group A (86 cases)	36	21	7	7	6	5	4
Group B (89 cases)	41	32	3	4	4	3	2
Statistical	5.645						
P value	0.464						

### Analysis of postoperative conditions

In the group with omental wrapping and modified pancreaticojejunostomy, there were 7 cases (8.1%) of grade B/C pancreatic fistula, 3 cases (3.5%) of biliary fistula, 1 case (1.2%) of delayed intra-abdominal haemorrhage and 1 case (1.2%) of reoperation; these rates were lower than those in the control group (17 cases (19.1%), 11 cases (12.4%), 8 cases (8.8%) and 7 cases (7.9%), respectively, P < 0.05). There were 2 cases (2.3%) of chyle fistula in the group covered with omentum and modified pancreaticojejunostomy, showing a lower rate than the 9 cases (10.1%) in the control group. The time until returning to a normal diet (6 d vs. 7 d), postoperative hospital stay (9 d vs. 10 d), and operation duration (5.8 h vs. 6.4 h) were all shorter in the group covered with omentum and modified pancreaticojejunostomy than in the control group (P < 0.05). The removal time of the pancreatic duct drainage tube in the omental wrapping and modified pancreaticojejunostomy group (13 d) was earlier than the extubation time of the control group (14 d) (P < 0.05). The removal time of the bile duct drainage tube in the omental wrapping and modified pancreaticojejunostomy group (14 d) was earlier than the time of extubation in the control group (17 d) (P < 0.05). However, in the 175 enrolled patients, there were no significant differences in complications such as abdominal infection and delayed gastric emptying between Groups A and B (P > 0.05). See Table 4 for details.

**Table 4:** Comparison of postoperative data between omentum liner and modified pancreaticojejunostomy group and control group

Group	B/C pancreatic fistula	Biliary fistula	Postoperative haemorrhage	Reoperation	Abdominal infection	Chylorrhea
	Example (%)	Example (%)	Example (%)	Example (%)	Example (%)	Example (%)
Group A (86 cases)	7 (8.1)	3 (3.5)	1 (1.2)	1 (1.2)	5 (5.8)	2 (2.3)
Group B (89 cases)	17 (19)	11 (12.4)	8 (8.8)	7 (7.9)	11 (12.4)	9 (10.1)
Statistical	4.315	4.677	5.491	4.504	2.256	4.502
P value	0.038	0.031	0.019	0.034	0.133	0.034
Group	Delayed gastric emptying	Open eating time	Postoperative hospitalization days	Removal time of pancreatic duct	Duration of surgery	Bile duct extraction time
	Example (%)	(day)	(day)	(day)	(hours)	(day)
Group A (86 cases)	2 (2.3)	6 (4–11)	9 (7–42)	13 (9–21)	5.8 (5.0–8.0)	14 (13–32)
Group B (89 cases)	5 (5.6)	7 (6–13)	10 (9–52)	14 (11–28)	6.4 (5.4–9.2)	17 (15–39)
Statistical	1.235	− 4.193	− 3.446	− 2.110	− 5.106	− 5.362
P value	0.267	0.000	0.001	0.035	0.000	0.000

### Analysis of postoperative inflammation index

None of the patients in either had a history of infection before the operation. In our study, the CRP, IL-6, and PCT values gradually decreased over time after the operation. The CRP, IL-6, and PCT levels decreased more in Group A than in Group B on the first, third, and seventh postoperative days, with P values less than 0.05, indicating that the difference is significant. See Table 5 for details.

**Table 5:** Comparison of postoperative inflammation index between two groups

Clinical indicators	Group A (n = 86)	Group B (n = 89)	Statistical	P value
CRP (mg/L)				
First postoperative day	13.05 ± 2.76	18.25 ± 1.64	10.937	0.023
Third postoperative day	12.56 ± 2.19	15.47 ± 2.03	11.217	0.017
Seventh postoperative day	7.66 ± 2.39	9.74 ± 1.93	6.542	0.004
IL-6 (pg/ml)				
First postoperative day	24.52 ± 4.05	32.16 ± 6.37	13.14	0.023
Third postoperative day	10.54 ± 3.15	19.21 ± 7.53	10.65	0.019
Seventh postoperative day	6.84 ± 2.25	8.22 ± 2.64	7.16	0.006
PCT (ng/ml)				
First postoperative day	0.125 ± 0.043	0.216 ± 0.076	12.66	0.045
Third postoperative day	0.089 ± 0.022	0.104 ± 0.043	13.27	0.028
Seventh postoperative day	0.037 ± 0.031	0.072 ± 0.028	8.56	0.013

### Univariate and multivariate analysis of risk factors for postoperative pancreatic fistula

Our univariate analysis revealed a correlation between the incidence of pancreatic fistula after pancreaticoduodenectomy and BMI ≥ 24 (HR, 3.54; 95% CI, 1.94–5.37; P = 0.019), pancreatic duct diameter < 3 mm (HR, 2.04; 95% CI, 1.23–3.68; P = 0.000), and no omental wrapping, and modified pancreaticojejunostomy (HR, 4.34; 95% CI, 2.26–6.73; P = 0.035). Multivariate logstic regression analysis was further performed using the risk factors determined significant by univariate analysis, and the results showed that BMI ≥ 24 (HR, 2.86; 95% CI, 1.85–4.26; P = 0.016), pancreatic duct diameter < 3 mm (HR, 1.85; 95% CI, 1.02–4.41; P = 0.007), and no omental wrapping, and modified pancreaticojejunostomy (HR, 4.02; 95% CI, 1.85–5.81; P = 0.010) were significant independent risk factors for pancreatic fistula after PD (P < 0.05). See Table 6 for details.

**Table 6:** Univariate and multivariate logistic regression analysis of risk factors of pancreatic fistula following pancreaticoduodenectomy

Variable	Univariate analysis			Multivariate analysis		
	HR	95%CI	P value	HR	95%CI	P value
Age (≥ 65 or < 65)	1.13	0.87–1.34	0.570			
Gender (female/ male)	0.83	0.42–1.32	0.981			
BM (≥ 24 or < 24)	3.54	1.94–5.37	0.019	2.86	1.85–4.26	0.016
Pancreatic duct diameter (mm, < 3 or ≥ 3)	2.04	1.23–3.68	0.000	1.85	1.02–4.41	0.007
Duration of operation (h, ≥ 6.5 or < 6.5)	1.27	0.87–1.54	0.405			
Intraoperative blood loss (ml, ≥ 1000 or < 1000)	1.03	0.15–1.47	0.656			
Wrapping with omentum and improving pancreaticojejunostomy (no/ yes)	4.34	2.26–6.73	0.035	4.02	1.85–5.81	0.010
Preoperative albumin (g/l, < 35 or ≥ 35)	1.08	1.01–1.97	0.065			
Bilirubin (umol/l, ≥ 34 or < 34)	2.42	1.21–4.17	0.819			
Postoperative pathology is benign and malignant (vicious/ good)	1.34	0.47–2.16	0.919			
Pancreatic texture (soft/firm)	0.58	0.23–2.19	0.274			

### Risk factors associated with overall survival

We also performed univariate Cox regression analysis to identify risk factors associated with overall survival after PD. Patient age ≥ 65 years (HR, 3.28; 95% CI, 2.17–5.34; P = 0.013), BMI ≥ 24(HR, 1.34; 95% CI, 1.21–4.29; P = 0.023), pancreatic duct diameter < 3 mm (HR, 1.04; 95% CI, 1.02–2.18; P = 0.010), no omental wrapping and, modified pancreaticojejunostomy (HR, 3.12; 95% CI, 1.21–4.64; P = 0.025), and malignant postoperative pathology (HR, 4.15; 95% CI, 2.47–6.12; P = 0.002) were risk factors associated with overall survival after PD. In multivariate analysis, a patient age ≥ 65 years (HR, 2.86; 95% CI, 2.03–5.06; P = 0.017), BMI ≥ 24(HR, 1.29; 95% CI, 1.15–3.96; P = 0.017), pancreatic duct diameter < 3 mm (HR, 1.25; 95% CI, 1.23–2.34; P = 0.027), no omental wrapping and, modified pancreaticojejunostomy (HR, 3.53; 95% CI, 1.52–4.41; P = 0.016), and malignant postoperative pathology (HR, 5.02; 95% CI, 2.36–5.84; P = 0.006) were identified as significant independent risk factors associated with overall survival after PD. See Table 7 for details.

**Table 7:** Predictive factors of overall survival of postoperative Pancreaticoduo-denectomy

Variable	Univariate analysis			Multivariate analysis		
	HR	95%CI	P value	HR	95%CI	P value
Age (≥ 65 or < 65)	3.28	2.17–5.34	0.013	2.86	2.03–5.06	0.017
Gender (female/ male)	0.764	0.33–2.12	0.665			
BMI (≥ 24 or < 24)	1.34	1.21–4.29	0.023	1.29	1.15–3.96	0.017
Pancreatic duct diameter (mm, < 3 or ≥ 3)	1.04	1.02–2.18	0.010	1. 25	1.23–2.34	0.027
Duration of operation (h, ≥ 6.5 or < 6.5)	2.37	0.68–3.48	0.842			
Intraoperative blood loss (ml, ≥ 1000 or < 1000)	1.56	0.95–3.37	0.556			
Wrapping with omentum and improving pancreaticojejunostomy (no/ yes)	3.12	1.21–4.64	0.025	3.53	1.52–4.41	0.016
Preoperative albumin (g/l, < 35 or ≥ 35)	1.53	1.41–2.67	0.074			
Bilirubin (umol/l, ≥ 34 or < 34)	2.23	1.31–4.25	0.319			
Postoperative pathology is benign and malignant (vicious/ good)	4.15	2.47–6.12	0.002	5.02	2.36–5.84	0.006
Pancreatic texture (soft/firm)	0.77	0.63–2.49	0.316			

## Discussion

According to worldwide cancer statistics in 2018, pancreatic cancer was the seventh leading cause of cancer death. It is estimated that pancreatic cancer will overtake breast cancer to become the third leading cause of cancer death in the future [28, 29]. Pancreatic cancer is a highly malignant tumour, and surgery is the only treatment with curative potential [29, 30]. The mortality rate after PD is less than 5% with surgical intervention [6], but pancreaticoduodenectomy remains a challenging procedure with a high incidence of complications. How to reduce the postoperative complications of PD and improve the prognosis of patients has become a focus of attention for pancreatic surgeons. The most common complications of PD include pancreatic fistula, delayed gastric emptying, and infection [31]. Pancreatic fistula and delayed gastric emptying have been shown to be the most significant postoperative complications of Whipple procedures [32]. In particular, the incidence of POPF remains as high as 20% [33]. POPF is one of the most serious complications after PD and increases the risk of other complications, delays hospital discharge and increases hospital costs [34, 35]. Improper management of pancreatic fistula will lead to abdominal infection, delayed haemorrhage, and even death in severe cases [36].

The normal pancreas is soft and fragile, and prone to bleeding during anastomosis. The risk of pancreatic fistula after anastomosis is also high. A fibrotic, firm pancreas lowers the difficulty of anastomosis. In this study, the pancreas was soft, and a firm texture (HR, 0.58; 95% CI, 0.23–2.19; P > 0.05) was not an independent risk factor for postoperative pancreatic fistula. It should be noted that whether the texture of the pancreas was soft or firm was determined by the operator during the operation, and there was no objective evaluation standard [37]. Activated pancreatic fluid is highly corrosive, and once pancreatic fistula occurs, it will lead to poor drainage in the operation area and accumulation of pancreatic fluid, leading to delayed haemorrhage after pancreatic surgery. Pancreatic fistula is the most important complication after PD. In different reports, the methods used vary. It has been proven in practice that methods such as placing a pancreatic duct support tube [38] and biological mesh[39] in the pancreaticojejunostomy does not have a substantive role in the prevention of pancreatic fistula. There are many types of pancreaticojejunostomy, including end-to-end intussusception anastomosis between the pancreas and jejunum, end-to-side anastomosis between the pancreas and jejunum, pancreaticojejunostomy between the mucosa of pancreatic duct and jejunum, binding pancreaticojejunostomy and pancreaticogastrostomy. However, there is no accepted anastomosis method that can completely avoid pancreatic fistula.

The greater omentum vessels are very rich, and omental wrapping around the pancreaticojejunostomy can improve the blood supply to the anastomosis and provide secretion, defence, a large area and strong absorption capacity [40]. The application of greater omental wrapping around the pancreaticojejunostomy in PD surgery can prevent the activation of pancreatic fluid and prevent the occurrence of pancreatic fistula [41, 42]. Modified pancreaticojejunostomy can avoid damage to the pancreatic tissue caused by excessive tension and does not affect the blood supply around the anastomosis. During the anastomosis process, the wrapping procedure is simple, the technical difficulty is low, and healing of the anastomosis can be facilitated. The patients in this retrospective study were grouped according to surgical approach and procedure. Different symptomatic treatments were adopted according to the patients' symptoms and test results before surgery to correct water and electrolyte disorders and anaemia. Vitamin K1 was used to improve coagulation function for patients with jaundice. However, whether it is necessary to reduce jaundice before surgery is still controversial [43]. Conservative treatment is the cornerstone of postoperative PF management [44]. In theory, the use of somatostatin can reduce the incidence of pancreatic fistula [45]. Patients in both groups received routine treatments for infection control, acid and enzyme inhibition, and nutrition support after surgery. The patients' vital signs and colour and volume of the drainage fluid were detected after the operation, and serum amylase and drainage fluid amylase were also examined. It has been reported that a serum amylase level 3 times higher than the upper limit of normal after the operation is an independent risk factor for POPF [46]. Postoperative statistics showed that the incidence of pancreatic fistula in the omental wrapping and modified pancreaticojejunostomy group (8.1%) was significantly lower than that in the control group (19%), and the incidence of complications such as biliary fistula and delayed intra-abdominal haemorrhage was also lower than that in the control group. Furthermore, the time until a return to a normal diet (6 d vs. 7 d), hospitalization duration (9 d vs. 10 d), time to removal of the pancreatic duct drainage tube (13 d vs. 14 d), and time to removal of the bile duct drainage tube (14 d vs. 17 d) were shorter in the group with omentum wrapping and modified pancreaticojejunostomy than in the control group. Of the 147 PD procedures performed by Shah [17], only 4% of the 101 patients who underwent omental wrapping had pancreatic fistula, while the incidence of pancreatic fistula was as high as 17.4% in the 46 patients without omental wrapping. In a systematic review of 4384 cases, Andreasi et al. [47] found that omental wrapping following pancreaticojejunostomy could significantly benefit patients by reducing the incidence of postoperative pancreatic fistula. Maeda et al. [48] found in a study of 100 PD patients with or without omental wrapping that although the incidence of pancreatic fistula did not decrease after omentum wrapping, it effectively reduced the risk of delayed abdominal bleeding. All these findings have fully indicated that wrapping the pancreaticojejunostomy with greater omentum can reduce the occurrence of pancreatic fistula or other complications to a certain extent. Omental wrapping and isolation play a positive role in the healing of pancreaticojejunostomy and the prevention of pancreatic fistula. Even if pancreatic fistula occurs, pancreatic fluid can be fully absorbed by the absorption function of the omentum. The greater omentum can also isolate blood vessels, such as the GDA stump, from the pancreaticojejunostomy to avoid delayed intraperitoneal haemorrhage caused by the erosion of blood vessels resulting from pancreatic fistula. Clinically correct and high-quality anastomosis can effectively reduce the occurrence of postoperative pancreatic fistula. Studies have shown that human omentum contains a large number of iNKT cells, which can control inflammation and tissue damage, with unique immune regulation and/or anti-metastasis function [49]. The pedicled omentum is an autologous tissue with abundant phagocytes and blood vessels that can effectively improve the blood supply to the anastomosis. Wrapping the anastomosis with omentum can not only reduce the occurrence of anastomotic leakage, but also promote the limitation of the leakage through inflammation and reduce the risk of secondary surgery. In this study, CRP, IL-6, and PCT were measured 1, 3, and 7 days after surgery. The patients in the free omental wrapping and modified pancreaticojejunostomy group had faster reductions in postoperative inflammatory indicators, and the differences were statistically significant. It was proven that the omentum can better control the postoperative inflammatory reaction, demonstrating the strong anti-inflammatory effect of the omentum itself.

Yap [50] used pancreaticogastrostomy (PG) in 47 patients and found PG to be a safe and effective anastomosis with fewer complications than pancreaticojejunostomy. It has been reported that modified pancreaticojejunostomy with double-layer pancreatic duct anastomosis can significantly reduce the incidence of POPF [51]. Modified pancreaticojejunostomy reduces the incidence of POPF and offers a new method of anastomosis for PD patients [52]. It has been reported that the use of modified purse-string sutures for pancreaticojejunostomy can reduce postoperative complications [53].Currently, there is still ongoing debate about which anastomosis is best. According to the authors, pancreatic surgeons must continually innovate anastomosis techniques to minimize surgical complications. To reduce the complications of pancreatic fistula, the traditional pancreaticojejunostomy method has been continuously improved by surgeons. In this study, modified pancreaticojejunostomy was used for pancreaticojejunostomy, i.e., end-to-side anastomosis of the broken end of the pancreas with the seromuscular layer of the jejunum. In contrast, traditional pancreaticojejunostomy requires mucosa-to-mucosa suture after seromuscular layer sutures,which greatly increases the duration of surgery. Modified pancreaticojejunostomy is simple and feasible, and it also reduces the incidence of blood supply disorders at the pancreaticojejunostomy site. In this retrospective study of 175 patients, the removal time of the pancreatic duct drainage tube and the bile duct drainage tube in the group with isolation and modified pancreaticojejunostomy surrounded by free omentum was earlier than that in the control group; moreover, the return to a normal diet occurred earlier, and the postoperative hospital stay was shorter. However, the incidences of intra-abdominal infection and delayed gastric emptying after surgery did not differ significantly between the two groups. We believe that the omental wrapping group generally had elderly patients with intraperitoneal infection, which might partly influence the statistical results. Abdominal infection may occur when pancreatic fistula develops to a more serious extent, and the omental wrapping technique and modified pancreaticojejunostomy can reduce the incidence of pancreatic fistula. It has been reported that any measure to reduce postoperative pancreatic fistula would reduce SSIs after pancreaticoduodenectomy [54]. Therefore, the omental wrapping technique for modified pancreaticojejunostomy also played a role in reducing the incidence of intra-abdominal infection. There was no significant difference between the two groups in delayed gastric emptying, and all patients were cured by nonsurgical conservative treatment.

Univariate analysis showed that the greater omental wrapping technique and modified pancreaticojejunostomy were all factors related to the occurrence of pancreatic fistula after PD, along with the patients’ BMI and width of the pancreatic duct. After modified pancreaticojejunal anastomosis, the greater omentum was spread behind the anastomosis, which reduced the incidence of pancreatic fistula. Omental wrapping isolates the anastomosis from the portal vein, mesenteric artery and vein, and other vessels to avoid intraperitoneal haemorrhage caused by corrosion of the blood vessels by pancreatic juice. Only one patient in the omental wrapping combined with modified pancreaticojejunostomy group underwent reoperation due to intra-abdominal haemorrhage, while seven patients in the control group required reoperation due to complications. The operation time of the patients in Group A was 5.8 h, which was shorter than the 6.4 h in Group B. The modified panceaticojejunostomy simplifies the manual anastomosis process, and further shortens the whole operation. The incidence of pancreatic fistula and other complications after wrapping the anastomosis with free omentum after modified pancreaticojejunostomy was lower than that in the control group. In a retrospective study of 900 PD patients who were excluded from total pancreatectomy, Fujii et al. [55] found that a BMI greater than 25 was the only factor for delayed healing of POPF. High BMI also increases the duration of PD surgery [56]. Tanaka et al. [57] believed that patients with POPF often had a higher BMI than those without POPF. Preoperative CT examination of patients revealed pancreatic fat infiltration, and POPF was found to be significantly associated with a high pancreatic fat rate. Measuring the patients’ pancreatic fat percentage with CT and evaluating the patients’ preoperative pancreatic features can effectively predict POPF. Braga [58] considered that increased BMI and the presence of fatty pancreas and pancreatic fibrosis were risk factors for pancreatic fistula after surgery, and based on these three factors, pancreatic fistula could be scored and predicted, allowing preventive measures to be formulated. It has been reported in the literature that in patients with diabetes, total pancreatic fat is significantly reduced, and fibrosis is aggravated, which actually plays a protective role for patients with POPF [59]. The univariate and multivariate regression analysis of BMI in 175 patients in this retrospective study revealed that BMI was an independent risk factor for pancreatic fistula. A pancreatic duct diameter less than 3 mm was also an independent risk factor for POPF. A retrospective study of 529 PD patients in the Department of Hepatobiliary and Pancreatic Surgery of Beijing Tumor Hospital showed that a main pancreatic duct size less than 3 mm was significantly associated with postoperative pancreatic fistula, and different pancreaticojejunostomy methods should be selected according to the different diameters of the pancreatic ducts [60]. It has been reported that POPF can be greatly reduced by minimizing intraoperative bleeding [61, 62]. In this study, pancreatic fistula occurred postoperatively in seven of the cases in which the intraoperative bleeding volume was greater than 1000 ml. Among the patients whose intraoperative blood loss volume was less than 1000 ml, 17 patients developed pancreatic fistula after the operation. After statistical analysis, intraoperative blood loss was not found to be a risk factor for pancreatic fistula. According to the postoperative univariate and multivariate analyses, no omental wrapping and the modified pancreaticojejunostomy technique were risk factors associated with postoperative pancreatic fistula and overall survival after pancreaticoduodenectomy. Therefore, the combined application of this technique can reduce complications such as pancreatic fistula, improve the overall survival rate, and benefit patients. In summary, the continuous optimization of large omentum pad technology and pancreaticojejunostomy plays an important role in reducing postoperative pancreatic fistula and other complications. Simple and feasible omental wrapping and modified pancreaticojejunostomy techniques shorten the operation duration and continuously benefit patients. It should be noted that during omental wrapping, special attention should be given to the blood supply of the selected omentum, and pedunculated omentum with a normal colour should be selected to avoid severe abdominal infection due to ischaemic and necrotic tissue. Additionally, the omental bleeding points must be strictly ligated before wrapping the pancreaticojejunostomy, and the integrity of the selected major vessels of the greater omentum must be ensured.

## Conclusions

The application of free greater omental wrapping and isolation tomodified pancreaticojejunostomy in pancreaticoduodenectomy can effectively reduce the occurrence of complications such as pancreatic fistula after surgery. Moreover, the greater omentum can also inhibit inflammatory reactions to a certain extent. This operation is simple and safe, reduces the occurrence of postoperative pancreatic fistula without increasing risk and is beneficial to patient prognosis, indicating that this approach is worthy of promotion in PD. This is a single-centre retrospective study. Due to the small sample size, there must be some bias factors. Large-sample multicentre studies are still needed to verify the efficacy of omental wrapping and modified pancreaticojejunostomy.

## Data Availability

The datasets of the current study are available from the corresponding author upon reasonable request.
